# MUTYH Deficiency Is Associated with Attenuated Pulmonary Fibrosis in a Bleomycin-Induced Model

**DOI:** 10.1155/2020/4828256

**Published:** 2020-10-16

**Authors:** Qingmin Sun, Jingwen Chen, Lizhi Xu, Jiaqi Kang, Xin Wu, Yan Ren, Yusaku Nakabeppu, Yaping Wang

**Affiliations:** ^1^Department of Medical Genetics, Nanjing University School of Medicine, Nanjing 210093, China; ^2^Department of Pharmacy, Jiangsu Province Hospital of Chinese Medicine, Affiliated Hospital of Nanjing University of Chinese Medicine, Nanjing 210029, China; ^3^Jiangsu Key Laboratory of Molecular Medicine, Nanjing University, Nanjing 210093, China; ^4^Division of Neurofunctional Genomics, Department of Immunobiology and Neuroscience, Medical Institute of Bioregulation, Kyushu University, Fukuoka 812-8582, Japan

## Abstract

Idiopathic pulmonary fibrosis (IPF) is a progressive, irreversible lung disease of unknown etiology with limited survival. IPF incidence and prevalence increase significantly with aging, which is associated with an age-related accumulation of oxidative DNA damage. The *Mutyh* gene is involved in the base excision repair (BER) system, which is critical for repairing the misincorporated adenine that is opposite to the oxidized guanine base, 8-oxoguanine, and maintaining the fidelity of DNA replication. We used *Mutyh* knockout mice and a bleomycin-induced pulmonary fibrosis model to test the effect of MUTYH deficiency on lesion progression. Unexpectedly, a much less severe lesion of pulmonary fibrosis was observed in *Mutyh*^−/−^ than in *Mutyh*^*+*/*+*^mice, which was supported by assay on protein levels of TGF-*β*1 and both fibrotic markers, *α*-SMA and Vimentin, in pulmonary tissues of the model animals. Mechanically, MUTYH deficiency prevented the genomic DNA of pulmonary tissue cells from the buildup of single-strand breaks (SSBs) of DNA and maintained the integrity of mtDNA. Furthermore, increased mitochondrial dynamic regulation and mitophagy were detected in pulmonary tissues of the bleomycin-induced *Mutyh*^−/−^ model mice, which could reduce the pulmonary epithelial cell apoptosis. Our results suggested that MUTYH deficiency could even induce protective responses of pulmonary tissue under severe oxidative stress.

## 1. Introduction

Idiopathic pulmonary fibrosis (IPF) is the most common form of idiopathic interstitial pulmonary disease, and it is characterized by extracellular matrix deposition, which leads to a progressive decline in lung function. IPF is an age-related fatal disease with a median survival of 2–5 years after diagnosis [1]. Although the development of IPF remains to be understood, increased oxidative stress and mitochondrial dysfunction have been considered to contribute to its pathogenesis [2].

Accumulating evidence shows that oxidative stress induces mitochondrial DNA (mtDNA) damage and plays a key role in pulmonary fibrosis and other age-related diseases. Mitochondrial-derived reactive oxygen species (ROS) trigger the mtDNA damage response and apoptosis of alveolar epithelial cells (AECs). Notably, 8-oxoguanine (8-oxoG) is a major ROS-induced oxidized base lesion in DNA, and its accumulation is associated with mitochondrial dysfunction and multiple organ injury [3, 4]. This lesion is mutagenic, and it is a major cause of G:C to T:A transversions in genomic DNA [5, 6]. Under physiological condition, the oxidized lesion can be corrected by the base excision repair (BER) systems in cells, which is initiated by DNA glycosylases that excise damaged bases and is critical for maintaining DNA replication fidelity [7]. Human MutY homolog (MUTYH) in the BER system identifies and removes adenine that is misincorporated opposite to 8-oxoG in template DNA, which prevents the base substitutions of G:C to T:A. Following excision of the mismatched adenine, an apurinic (AP) site is left on the DNA strand and generates a single-strand break (SSB), which is further repaired [8]. During severe oxidative stress, SSBs in genomic DNA may accumulate and initiate cell death via the BER process [9, 10]. Moreover, the germline mutation of *MUTYH* and increased mutagenesis in genomic DNA have been identified as the cause for autosomal recessive familial adenomatous polyposis, known as MUTYH-associated polyposis [11, 12].

However, there are some contradictions in the functional consequences of *Mutyh* knockout in model mice [7, 10, 13, 14], which indicates that the degree and duration of oxidative stress result in different consequences during MUTYH deficiency. We previously investigated the histopathological changes of multiple organs in *Mutyh*^−/−^ mice with D-galactose (D-gal) exposure-induced oxidative stress and found that MUTYH deficiency caused more pronounced damage in cardiac, hepatic, and renal tissues but less damage in pulmonary tissues [14], which inspired us to investigate whether MUTYH deficiency affected pulmonary fibrogenesis. The current study compared bleomycin-induced pulmonary fibrosis between *Mutyh*^−/−^ and wild-type mice and demonstrated that MUTYH deficiency resulted in an attenuated fibrosis phenotype due to the alleviation of SSB accumulation in genomic DNA and reduced epithelial cell apoptosis. These results provide novel insight into the protection of pulmonary tissue under severe oxidative stress.

## 2. Materials and Methods

### 2.1. Animals and Induction of the Pulmonary Fibrosis Model

**Figure 1:**
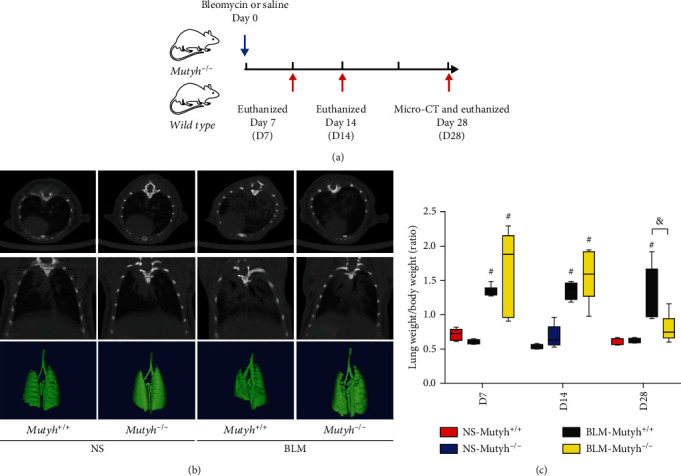
The bleomycin- (BLM-) induced pulmonary fibrosis mouse model was evaluated using Micro-CT and lung weight/body weight ratios. (a) Schematic representation of the experimental design is shown. (b) Micro-CT and 3D digital reconstruction were employed to directly observe the fibrotic lesions in the lungs of mice on day 28 after intratracheal administration with a single dose of BLM or normal saline (NS) on day 0. (c) Lung weight/body weight ratios are shown compared to NS groups. BLM treatment markedly increased the ratios at all three time points. On day 28, BLM-*Mutyh*^−/−^ mice exhibited a significantly lower ratio of lung weight/body weight, compared to BLM-*Mutyh*^+/+^ mice (*n* ≥ 5, *P* = 0.006). Statistical significance was analyzed using two-way ANOVA and followed by the LSD post hoc test. ∗ represents *P* < 0.05 and # represents *P* < 0.01 compared with the NS-*Mutyh*^+/+^ group. & represents *P* < 0.01 compared to the BLM-*Mutyh*^+/+^ group.

Wild-type (C57BL/6J) mice were purchased from the Model Animal Research Center of Nanjing University. *Mutyh*^+/−^ mice were previously established and backcrossed to C57BL/6J mice as described previously [15], and *Mutyh*^−/−^ mice were obtained via inbreeding. Eight-week-old male mice (18-22 g) were used in the present experiment. All animals were maintained under standard conditions of 50% relative humidity, 21 ± 2°C, and a 12 h light cycle. To induce pulmonary fibrosis, 4 mg/kg bleomycin (BLM, Nippon Kayaku Co., Tokyo, Japan) was instilled intratracheally in a single dose, and an equal volume of normal saline (NS) was used as a control. The experimental mice were euthanized on days 7 (D7), 14 (D14), and 28 (D28) after intratracheal instillation. Lung tissues and serum from wild-type and *Mutyh*^*−*/*−*^ mice were collected for further analysis (Figure 1(a)). The animal experiments were performed based on the Jiangsu Provincial Experimental Animal Manage Committee under Contract 2011-0069, and the Experimental Animal Ethics Committee of Nanjing University approved the experimental protocols.

### 2.2. Histopathology, Immunohistochemistry, Immunofluorescence, and Electron Microscopy

For histological evaluation, hematoxylin and eosin (HE) staining was used for inflammatory responses and structural observations, and Masson's trichrome was used for the detection of collagen deposits. The lung tissues were fixed in 10% neutralized formaldehyde and embedded in paraffin for pathological examination. Sections (4 *μ*m) of lung tissues were deparaffinized, rehydrated, and sequentially stained with HE or Masson's trichrome. Morphological changes in interstitial fibrosis were scored separately using the Ashcroft method, as previously described [16]. Immunohistochemical (IHC) staining was performed to detect 8-oxoG using a monoclonal antibody against 8-oxoG (ab62623, Abcam, UK; 1 : 100) and enhanced using goat anti-mouse polyclonal horseradish peroxidase- (HRP-) conjugated IgG. The intensity of 8-oxoG immunostaining was quantified using the integrated optical density (IOD) value. Immunofluorescence studies used an anti-ssDNA antibody (1 : 100, 18731, Immuno-Biological Laboratories) or anti-SP-C antibody (1 : 2000, ab90716, Abcam Inc.), which was incubated with the sections at 4°C overnight. Sections were washed in PBS, and Alexa Fluor 488-conjugated goat anti-mouse/rabbit IgG (Invitrogen) was used as the secondary antibody. Nuclei were counterstained with DAPI (Sigma). Fluorescent images were captured using an Olympus fluorescence microscope (Olympus). Four nonoverlapping tissue fields (under ×40 magnification) were randomly selected in each section to evaluate fluorescent areas using ImageJ software (NIH, Bethesda, MD).

For transmission electron microscopy, lung tissue was fixed with 2.5% glutaraldehyde in 0.1 M sodium cacodylate buffer at pH 7.4 for examination in a transmission electron microscope system (Hitachi Regulus 8100). At least 5-10 cells from low-magnification images (×10,000) were used to quantitate the number of mitochondria per AECII (confirmed by the presence of lamellar bodies). A total of 70–100 individual mitochondria were used to determine the mitochondrial area. All mitochondrial morphometric analyses were performed using ImageJ software.

### 2.3. Cell Culture and *In Vitro* Transfection

A549 cell lines were purchased from ATCC (American Type Culture Collection, Manassas, VA, USA). A549 cells were cultured in 1640 medium (Gibco) containing heat-inactivated 10% fetal bovine serum (FBS) and maintained in a humidified atmosphere of 95% air and 5% CO_2_ at 37°C. For siRNA transfection, A549 cells were seeded at 5 × 10^5^ cells/well in 6-well plates one day prior to transfection. siRNA transfection was performed when the cell density reached 60%-70% using Lipofectamine 3000 according to the manufacturer's protocol (Invitrogen, Thermo Scientific, MA, USA). Three siRNA duplexes targeting MUTYH (siRNA-1, siRNA-2, and siRNA-3) were chemically synthesized and ordered from GenePharma (Shanghai, China). A scrambled siRNA (GenePharma, China) was also provided as a negative control. The sequences of the siRNAs are listed in Supplementary Table S1.

### 2.4. Hydroxyproline Assay

Hydroxyproline (HYP) in lung tissues was quantified to evaluate collagen content using a kit purchased from Jiancheng Biotechnology Institute (Nanjing, China). The HYP assay was performed according to the manufacturer's protocol.

### 2.5. Quantitative RT-PCR

Total RNA was extracted from A549 cells using TRIzol Reagent (Invitrogen) following the manufacturer's protocol. MUTYH and *β*-actin (control) mRNA levels were assessed using SYBR Green Quantitative PCR. Reactions were performed according to the manufacturer's instructions using Power SYBR Green PCR Master Mix (Applied Biosystems Inc., USA). The primer sequences for PCR are listed in Supplementary Table S2. Quantitative PCR was performed using the ABI Prism 7300 sequence detection system (Applied Biosystems Inc., USA).

### 2.6. Long-Range Quantitative PCR Assay and Quantification of mtDNA

Long-range quantitative PCR assays were performed to assess nuclear DNA (nDNA) and mitochondrial DNA (mtDNA) integrity. Total DNA was extracted from the lung tissue of mice using the TIANamp Genomic DNA kit (TIANGEN Biotech, China) according to the recommended protocol. DNA was diluted, and the relative amounts of mtDNA/nDNA were quantified as previously described [12]. Long-range PCR assays were performed for a 10 kb fragment of mtDNA and an 8.7 kb fragment of *globin* for nDNA. The amplification protocol was performed using the following parameters: 95°C for 3 minutes; 95°C for 30 seconds, 62°C for 30 seconds, and 68°C for 10 minutes for 26 cycles (10 kb) or 35 cycles (8.7 kb); and a final extension for 10 minutes at 72°C. All products were quantified using Quant-iT PicoGreen dsDNA Reagent (Invitrogen, USA). The PCR products were assessed using 0.8% agarose gel electrophoresis to verify the target product.

For the quantification of mtDNA, a 117 bp mtDNA fragment was employed to obtain mtDNA content. Relative quantification of mtDNA content was determined based on the ratio of the 117 bp mtDNA fragment to the nuclear-encoded *β*-actin. The primers are listed in Supplementary Table S2. Quantification of mtDNA was performed using the ViiA 7 Real-Time PCR Systems (Applied Biosystems Inc., USA).

### 2.7. Western Blot and Apoptosis Analysis

Lung tissues from experimental mice and A549 cells were lysed in a radioimmune precipitation buffer. Proteins were separated using 10% SDS-PAGE and transferred to PVDF membranes, which were incubated with antibodies against *α*-SMA (Cell Signaling Technology, no. 19245), Vimentin (Cell Signaling Technology, no. 5741), TGF-*β*1 (R&D Systems, MAB240), E-cadherin (Cell Signaling Technology, no. 3195), Bax (Cell Signaling Technology, no. 5023), Cleaved-caspase-3 (Cell Signaling Technology, no. 9661), MFN2 (Cell Signaling Technology, no. 9482), DRP1 (Abcam, ab180769), PINK (Abcam, ab23707), and *β*-actin (Cell Signaling Technology, no. 8457). Protein bands were visualized using an enhanced chemiluminescence (ECL) kit and quantitated using ImageJ software. For apoptosis analysis, the cells were measured using an Annexin V-FITC/PI Apoptosis Detection Kit (KeyGen Biotech Co., Nanjing, China) and flow cytometry (BD Biosciences) according to the manufacturer's protocol.

### 2.8. Statistical Analysis

All values are described as the mean ± SEM of at least three independent experiments. GraphPad Prism 7.0 software (GraphPad Software Inc., San Diego, CA, USA) was used for plotting and statistical analyses. We performed a one-way or two-way ANOVA and post hoc LSD test when three or more groups were included. *P* values < 0.05 were considered statistically significant.

## 3. Results

### 3.1. MUTYH-Deficient Mice Showed Attenuated Pulmonary Fibrosis in a Bleomycin- (BLM-) Induced Model

To test whether MUTYH-deficient mice were more prone to pulmonary fibrosis, *Mutyh* knockout (*Mutyh*^−/−^) and wild-type (*Mutyh*^*+*/*+*^) mice were subjected to intratracheal administration of BLM to induce pulmonary fibrosis. These mice were divided into different groups and euthanized on day 7 (D7), 14 (D14), or 28 (D28) after the intratracheal administration of a single dose of BLM, which corresponded to the inflammation phase, transitional phase of inflammation/fibrosis, and late stage with increased deposition of lung collagen, respectively (Figure 1(a)). Chest microcomputed tomography (Micro-CT) was performed on mice in the D28 group. The results on CT imaging and three-dimensional digital reconstruction showed that a characteristic fibrosis lesion appeared in the lungs of mice with BLM treatment, both in *Mutyh*^−/−^ and *Mutyh*^*+*/*+*^mice, indicating that the pulmonary fibrosis was established successfully (Figure 1(b)). Unexpectedly, a much less severe pulmonary fibrosis image was observed in *Mutyh*^−/−^ than in *Mutyh*^*+*/*+*^ mice. We checked the ratios of lung weight/body weight and found an increased ratio among the BLM-treated mice when compared to their counterpart controls (normal saline (NS)) (Figure 1(c), *P* < 0.010 for each group). However, the ratio of *Mutyh*^*+*/*+*^mice of the D28 group was higher than that of *Mutyh*^−/−^ mice of D28 (*P* = 0.006), supporting the result observed on Micro-CT images.

**Figure 2:**
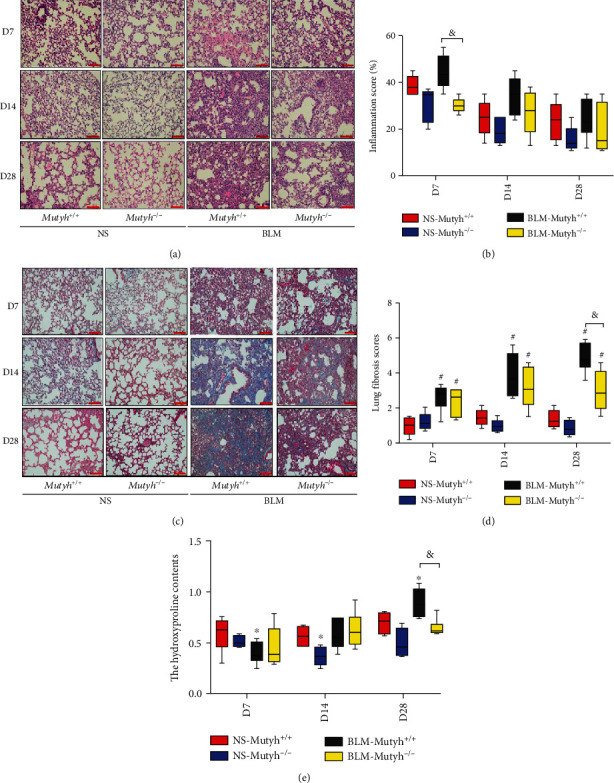
MUTYH-deficient mice exhibited attenuated pulmonary fibrosis in the BLM-induced model. (a) Representative histological sections of pulmonary tissues were obtained from experimental animals and subjected to HE staining. (b) The inflammatory score based on HE staining in the BLM-*Mutyh*^−/−^ group was significantly lower than that in BLM-*Mutyh*^+/+^ among D7 mice (*P* = 0.003). (c, d) Representative images of Masson's trichrome staining in pulmonary tissue sections revealed significantly decreased collagen deposition (blue) in BLM-*Mutyh*^−/−^ compared to BLM-*Mutyh*^+/+^ mice among the D28 group (*P* < 0.001). Scale bars: 100 *μ*m. (e) Collagen deposition was assessed by hydroxyproline contents. BLM-*Mutyh*^−/−^ exhibited significantly lower contents compared to BLM-*Mutyh*^+/+^ mice among the D28 group (*P* = 0.004). *n* ≥ 5 mice per group. Statistical significance was analyzed using two-way ANOVA and followed by the LSD post hoc test. ∗ represents *P* < 0.05 and # represents *P* < 0.01 compared with NS-*Mutyh*^+/+^ mice. & represents *P* < 0.01 compared to the BLM-*Mutyh*^+/+^ group.

Histological observations revealed increased inflammatory responses and an obvious deposition of collagen with the destruction of pulmonary tissue architecture in BLM-treated mice (Figure 2). The inflammatory score was higher in BLM-*Mutyh*^+/+^ D7 mice than BLM-*Mutyh*^−/−^ mice (Figures 2(a) and 2(b), *P* = 0.003). A denser collagen deposition was also observed in the pulmonary tissues of BLM-*Mutyh*^+/+^ of D28 mice than BLM-*Mutyh*^−/−^ D28 (*P* < 0.001) (Figures 2(c) and 2(d)). Consistently, the hydroxyproline content of pulmonary tissues and serum TGF-*β*1 level of BLM-*Mutyh*^*+*/*+*^ mice were higher than those of BLM-*Mutyh*^−/−^ among the D28 group (Figure 2(e), *P* = 0.004, Supplementary Fig. S1, *P* < 0.001).

### 3.2. MUTYH Deficiency-Associated Reduction of TGF-*β*1 and Fibrotic Marker Expression Mainly Occurred in the Late Stage of Fibrogenesis

**Figure 3:**
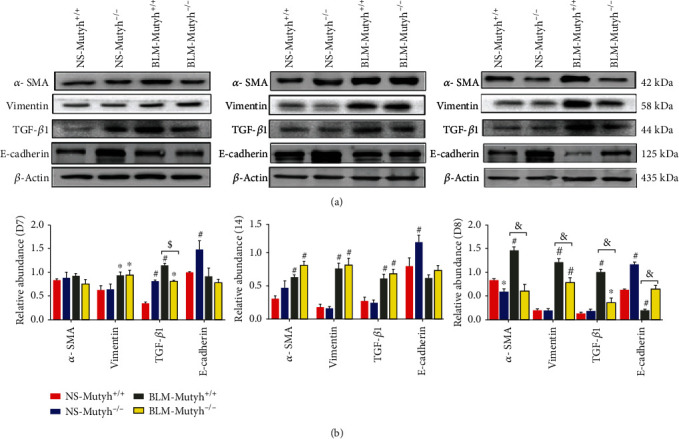
*Mutyh* ^−/−^-associated reduction of TGF-*β*1 and fibrotic marker expression in BLM-induced model mice. (a) Western blot showed an increased expression of TGF-*β*1 and fibrotic markers in pulmonary tissues from BLM-treated mice, especially in D14 and D28. In D28, MUTYH deficiency significantly reduced the levels of *α*-SMA, Vimentin, and TGF-*β*1 compared to BLM-*Mutyh*^+/+^ mice, while an upregulation expression of E-cadherin was observed in NS-*Mutyh*^−/−^ mice of all three groups and in BLM-*Mutyh*^−/−^ of D28. (b) Histograms of the protein measure show the relative expression of each protein (*n* = 3). Statistical significance was analyzed using two-way ANOVA and followed by the LSD post hoc test. ∗ represents *P* < 0.05 and # represents *P* < 0.01 compared to the NS-*Mutyh*^+/+^ group. $ represents *P* < 0.05 compared to the BLM-*Mutyh*^+/+^ group. $ represents *P* < 0.05 and & represents *P* < 0.01 compared to the BLM-*Mutyh*^+/+^ group.

TGF-*β*1 has been well established as a key profibrotic mediator in fibrotic diseases [17]. Next, we assessed the expression of TGF-*β*1 and the fibrotic markers, *α*-smooth muscle actin (*α*-SMA) and Vimentin, with Western blot. Compared to NS-*Mutyh*^+/+^ mice, TGF-*β*1 expression in pulmonary tissues was upregulated in the D7 group of NS-*Mutyh*^−/−^, BLM-*Mutyh*^*+*/*+*^, and BLM-*Mutyh*^−/−^ mice. Moreover, the expression levels of TGF-*β*1 in D7 mice of BLM-*Mutyh*^*+*/*+*^ were significantly higher than those in the counterpart of BLM-*Mutyh*^-/-.^ In D14 and D28, an obviously increased level of TGF-*β*1 was associated with bleomycin exposure. However, a significant reduction in TGF-*β*1 was observed in D28 BLM-*Mutyh*^−/−^ mice compared to BLM-*Mutyh*^+/+^ mice (Figure 3). Serum TGF-*β*1 levels in D28 BLM-*Mutyh*^−/−^ mice were significantly lower than those in D28 BLM-*Mutyh*^+/+^ mice (Supplementary Fig. S1). Moreover, the expression of *α*-SMA and Vimentin was lower in D28 mice of BLM-*Mutyh*^−/−^ than the counterpart of BLM-*Mutyh*^+/+^ (Figure 3). Interestingly, E-cadherin, an epithelial marker, always showed a higher expression in NS-*Mutyh*^−/−^ mice than NS-*Mutyh*^+/+^ mice at all three measured time points (*P* = 0.003, *P* = 0.020, and *P* < 0.001, respectively). This upregulation expression of E-cadherin was also observed in D28 mice of BLM-*Mutyh*^−/−^ when compared with D28 BLM-*Mutyh*^+/+^ (*P* = 0.004) (Figure 3). These results indicated that the MUTYH deficiency-associated alleviation of pulmonary fibrosis may be due to the reduced TGF-*β*1 signal in the late stage of BLM-induced fibrogenesis.

### 3.3. MUTYH Deficiency Had No Obvious Impact on 8-oxoG Levels but Significantly Reduced SSB Formation in the Pulmonary Tissues of BLM-Treated Mice

**Figure 4:**
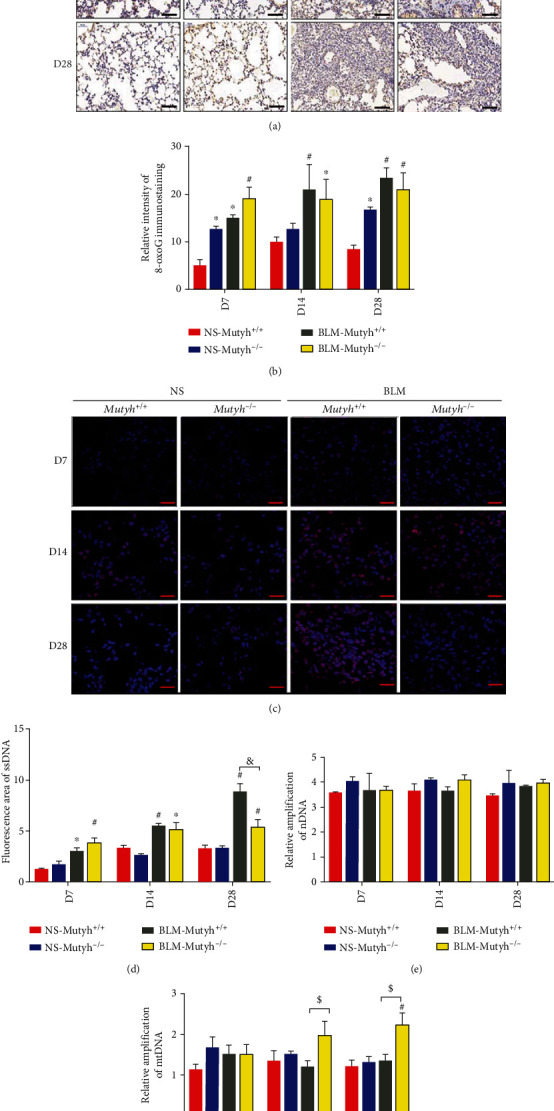
MUTYH deficiency alleviated SSB formation and maintained mtDNA integrity. (a, b) Representative images of immunostaining of 8-oxoG and quantification of the intensity of 8-oxoG immunostaining in pulmonary tissue sections (*n* = 4 for per genotype/treatment; scale bar: 100 *μ*m). (c, d) Pulmonary tissue specimens of mice were subjected to immunofluorescence staining with anti-ssDNA (red, original magnification: ×630, *n* = 4, and scale bar: 67 *μ*m). The immune complexes and DAPI-stained nuclei (blue) were visualized under a fluorescence microscope. Total fluorescence intensity of 4 fields selected randomly in 3 sections was quantified with ImageJ (b). (e, f) An 8.7 kb fragment from *β*-*globin* loci and a 10 kb fragment from mtDNA were used for measuring nDNA and mtDNA integrity, respectively. At least two independent experiments were performed for each sample (*n* ≥ 5). Statistical significance was analyzed using two-way ANOVA and followed by the LSD post hoc test. ∗ represents *P* < 0.05 and # represents *P* < 0.01 compared with the NS-*Mutyh*^+/+^ group. $ represents *P* < 0.05 compared to the BLM-*Mutyh*^+/+^ group. $ represents *P* < 0.05 and & represents *P* < 0.01 compared to the BLM-*Mutyh*^+/+^ group.

We performed immunohistochemistry to assess the 8-oxoG lesion resulting from oxidative DNA damage in the pulmonary tissue of model animals. The results showed that BLM treatment obviously elevated the 8-oxoG lesion either in *Mutyh*^−/−^ or *Mutyh*^+/+^ mice but no significant difference was observed between two genotype mice with the same administration except for saline control of the D7 and D28 groups (Figure 4(a)). These results indicate that MUTYH deficiency had little additional impact on BLM-induced oxidative DNA damage. Considering that functional MUTYH can initiate BER-associated accumulation of single-strand breaks (SSBs) in genomic DNA with BLM treatment, we further employed immunofluorescence to detect the single-stranded DNA (ssDNA) in the pulmonary tissues of model animals. As expected, the immunoreactivities for ssDNA were significantly higher in the mice treated with BLM than their counterparts receiving saline and in time dependence within our observation period after BLM intratracheal administration (Figure 4(c)). Moreover, a higher level of ssDNA accumulation was observed in pulmonary tissue of D28 mice of BLM-*Mutyh*^+/+^ than BLM-*Mutyh*^−/−^ (Figures 4(c) and 4(d), *P* < 0.001), suggesting that MUTYH-mediated BER would promote the SSB formation. Given that SSBs can be generated in the process of DNA lesion repair under oxidative stress and impair DNA integrity, therefore, we performed long-range PCR to determine the integrity of nDNA and mtDNA in the pulmonary tissues of animals with or without BLM-induced pulmonary fibrosis.  Figure 4(e) shows no differences in the levels of the long-range PCR products from nDNA between groups with different genotypes and/or administrations. However, there was an obvious difference in the levels of the long-range PCR products from mtDNA (Figure 4(f)). The relative amplification of mtDNA was significantly greater in BLM-*Mutyh*^−/−^ mice than BLM-*Mutyh*^*+*/*+*^ mice in the D14 and D28 groups (*P* = 0.015 and *P* = 0.011, respectively), which indicates that MUTYH deficiency was instrumental in maintaining the integrity of mtDNA following BLM treatment. There were no significant differences in mtDNA content in pulmonary cells between D14 and D28 BLM-*Mutyh*^+/+^ and BLM-*Mutyh*^−/−^ mice (Supplementary Fig. S2).

### 3.4. Apoptosis of Epithelial Cells Was Decreased in BLM-Treated *Mutyh*^−/−^ Mice

**Figure 5:**
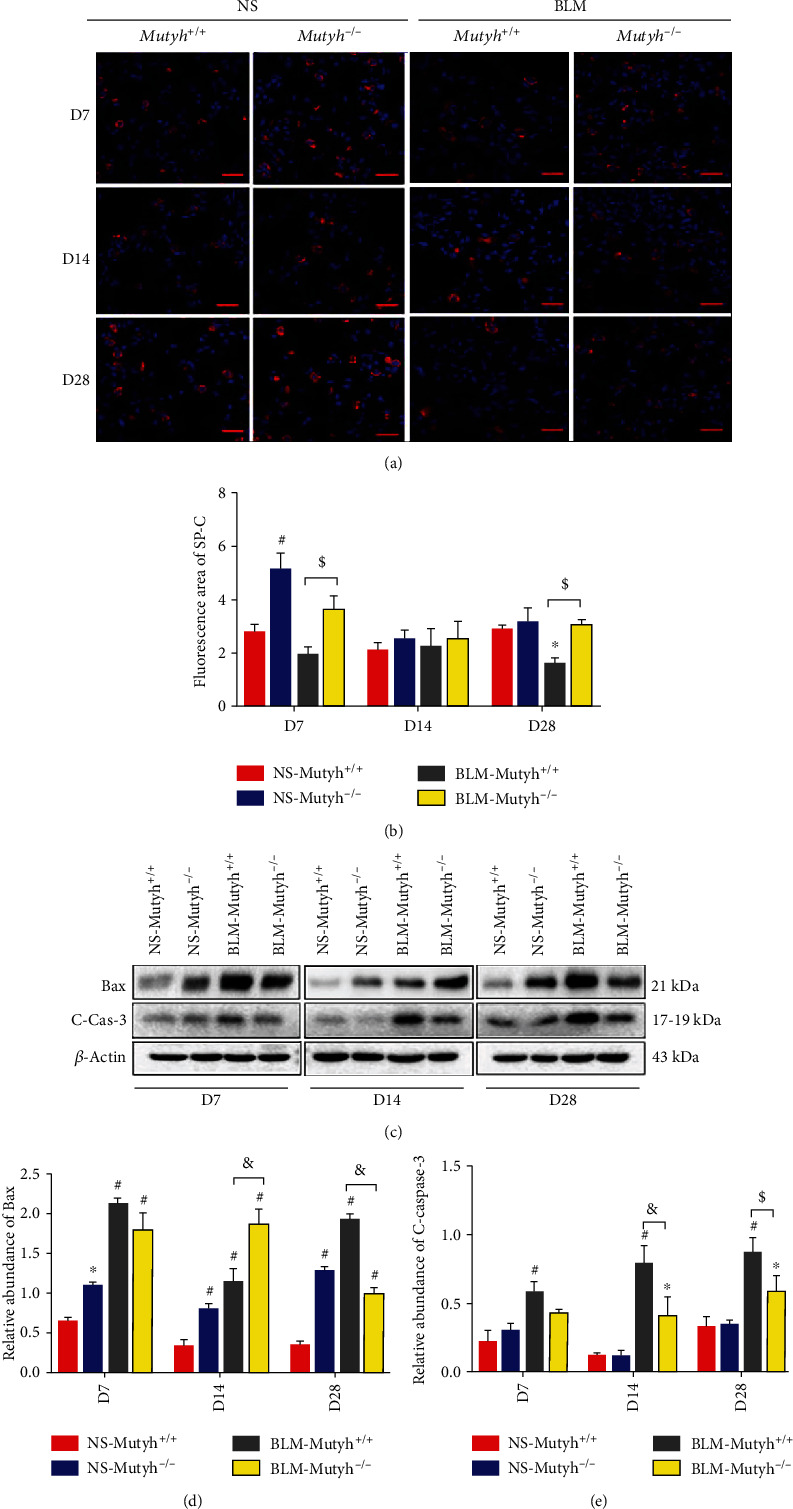
Apoptosis of epithelial cells was suppressed in *Mutyh*^−/−^ mice treated with BLM. (a) Pulmonary tissue sections of mice were subjected to immunofluorescence staining with anti-SP-C (red, original magnification: ×630, *n* = 4, and scale bar: 67 *μ*m). Merged images with DAPI-stained nuclei were shown. (b) Total fluorescence intensity of SP-C was quantified with ImageJ. (c) Protein extracts from pulmonary tissues of different groups were subjected to Western blotting with anti-Bax and Cleaved-caspase-3. (d, e) Histograms of the protein measure show the relative expression of each protein (*n* = 3). Statistical significance was analyzed using two-way ANOVA and followed by the LSD post hoc test. ∗ represents *P* < 0.05 and # represents *P* < 0.01 compared with the NS-*Mutyh*^+/+^ group. $ represents *P* < 0.05 and & represents *P* < 0.01 compared to the BLM-*Mutyh*^+/+^ group.

Mitochondrial dysfunction and alveolar epithelial cell type II (AECII) apoptosis have been recognized as an important role in the pathogenesis of pulmonary fibrosis [18]. We measured the area covered by AECIIs with immunofluorescence staining of surfactant protein C (SP-C) in pulmonary sections from each group of model animals. Interestingly, a larger SP-C positive area was observed in the pulmonary tissues of BLM-*Mutyh*^−/−^ mice than BLM-*Mutyh*^+/+^ of the D7 group (*P* = 0.012). A similar result was also found in D28 mice (*P* = 0.031), which suggested that MUTYH deficiency could be beneficial to AECII survival with BLM treatment (Figures 5(a) and 5(b)). The proapoptosis protein Bax and Cleaved-caspase-3 were also examined, and the results showed that MUTYH deficiency was associated with a decreased expression of Bax and Cleaved-caspase-3 in pulmonary tissues of mice treated with BLM in the D28 group (Figures 5(c)–5(e)).

### 3.5. Inhibition of MUTYH Expression Alleviated A549 Apoptosis *In Vitro* under Oxidative Stress

A549 cells (commonly used as a model of human alveolar type II pulmonary epithelium) were employed to test the effect of MUTYH deficiency on apoptosis *in vitro*. We chemically synthesized specific siRNAs (siRNA-1, siRNA-2, siRNA-3) targeting *MUTYH* and found that siRNA-2 significantly inhibited MUTYH expression at RNA and protein levels (Supplementary Fig. S3). Given that TGF-*β*1 has a role in triggering an epithelial cell apoptosis response, we examined the effects of MUTYH knockdown on the TGF-*β*1-induced apoptosis in A549 cells *in vitro*. We found that proapoptosis protein expression induced by TGF-*β*1 was inhibited with *MUTYH* siRNA treatment (Supplementary Fig. S4a). Furthermore, we used flow cytometry to investigate the effect of MUTYH knockdown on the apoptotic response of A549 cells under oxidative stress induced by H_2_O_2_ and menadione exposure. The results also showed that siRNA-2 significantly suppressed the apoptotic response and reduced the number of apoptotic cells (Supplementary Fig. S4b-e), indicating that knockdown of MUTYH expression could alleviate apoptosis under oxidative stress.

### 3.6. MUTYH Deficiency Contributes to Maintaining Mitochondrial Homeostasis under Severe Oxidative Stress

**Figure 6:**
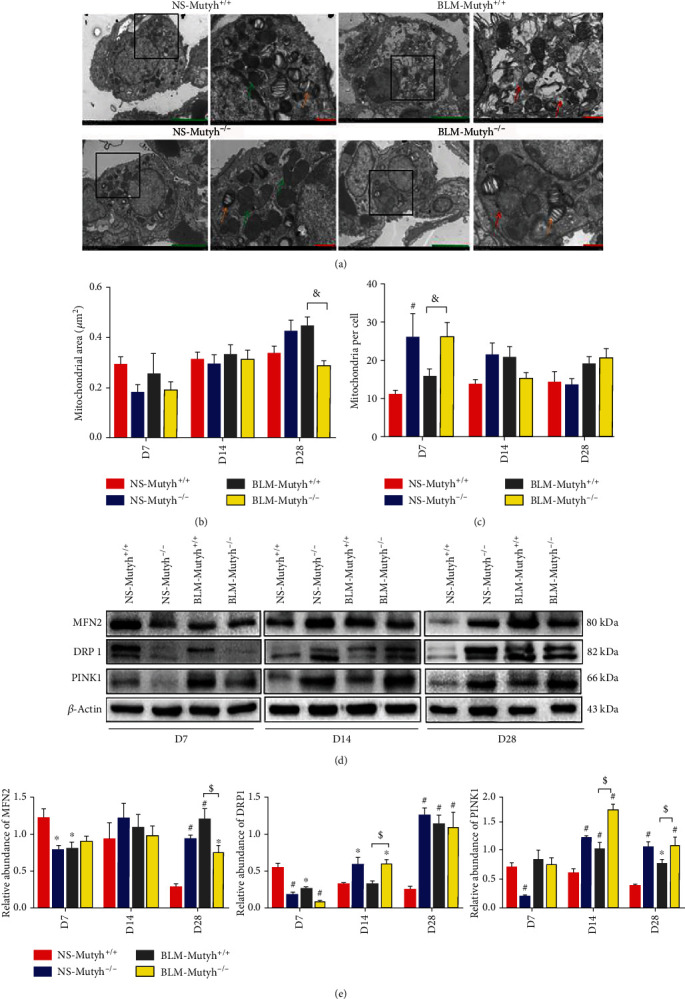
MUTYH deficiency maintained mitochondrial homeostasis. (a) Representative images of transmission electron microscopy (TEM) of lung AECIIs from different groups of mice (lamellar bodies used as identification marks, orange arrows; green scale bar: 5 *μ*m, *n* = 4). The boxed region of the image is magnified in the right panel (red scale bar: 1 *μ*m). Mitochondria in AECIIs of pulmonary tissues from model mice were mostly enlarged. The normal mitochondria were indicated with green arrows and dysmorphic ones with red arrows. (b) Quantitative morphometric analyses of the mitochondrial area in single AECII with TEM images. (c) Mitochondrial number per AECII was counted in the TEM images from different groups of mice. (d) Expression levels of the proteins involved in mitochondrial fusion/fission dynamic regulation. Western blotting with anti-MFN2, DRP1, and PINK1 was shown. (e) Histograms show the relative expression levels of MFN2, DRP1, and PINK1 proteins (*n* = 3). Statistical significance was analyzed using two-way ANOVA and followed by the LSD post hoc test. ∗ represents *P* < 0.05 and # represents *P* < 0.01 compared with the NS-*Mutyh*^+/+^ group. $ represents *P* < 0.05 and & represents *P* < 0.01 compared to the BLM-*Mutyh*^+/+^ group.

Next, we investigated the changes in mitochondrial homeostasis of AECIIs in pulmonary tissues of BLM-treated mice. The ultrastructural analyses with transmission electron microscopy (TEM) revealed that the enlarged mitochondria and increased mitochondrial area were evident in BLM-*Mutyh*^+/+^ mice compared to BLM-*Mutyh*^−/−^ among the D28 group (*P* = 0.001) (Figures 6(a) and 6(b)). BLM-*Mutyh*^−/−^ mice had more mitochondria in AECIIs than BLM-*Mutyh*^+/+^ mice in the D7 group (*P* = 0.010) (Figure 6(c)), while the swollen mitochondria were associated with BLM-induced pulmonary fibrogenesis in *Mutyh*^+/+^mice. Mitochondrial dynamic regulation of fusion, fission, and mitophagy contributes to mitochondrial quality control and functional stability. Therefore, we examined the expression of proteins involved in fusion, fission, and mitophagy, MFN2, DRP1, and PINK1, respectively, in pulmonary tissues of BLM-treated mice. Western blot analysis showed an upregulated expression of the mitochondrial fission protein DRP1 in BLM-*Mutyh*^−/−^ on D14 (*P* = 0.045) and inhibition of the fusion modulator MFN2 on D28 compared to BLM-*Mutyh*^+/+^ mice (*P* = 0.020) (Figures 6(d) and 6(e)). PINK1 is an important regulatory protein of mitochondrial morphology, and it showed significantly increased expression in BLM-*Mutyh*^−/−^ mice in the D14 (*P* < 0.001) and D28 groups (*P* = 0.049), which indicates that MUTYH deficiency contributes to the maintenance of mitochondrial dynamic regulation in mice with BLM-induced pulmonary fibrosis (Figures 6(d) and 6(e)).

## 4. Discussion

IPF is an age-related disease in which oxidative stress and mitochondrial dysfunction have been demonstrated to be involved in IPF pathogenesis [18]. As a critical member of the BER system, MUTYH deficiency could logically be considered to aggravate oxidative damage and increase the risk for IPF. Contrary to this expectation, our results showed an attenuated pulmonary fibrosis phenotype in *Mutyh*^−/−^ mice with BLM treatment, especially in the late stage of fibrogenesis (D28 group). Supporting data were also obtained from the measurement of protein expression of the important profibrotic cytokine, TGF-*β*1, fibrotic markers, *α*-SMA and Vimentin, and epithelial marker, E-cadherin, in the pulmonary tissues of the model animals. These differential responses related to *Mutyh* genotypes in pulmonary tissue likely occurred during the inflammation stage following BLM intratracheal instillation. The *Mutyh*^−/−^mice showed a much less severe inflammatory response when compared to the wild type among the D7 group. We further demonstrated an increased mitochondrial dynamic regulation and mitophagy in pulmonary tissues of *Mutyh*^−/−^ mice with BLM exposure, along with a reduced SSB formation and AECII apoptosis, which could contribute to reduced pulmonary fibrosis in *Mutyh*^−/−^ mice.

The DNA glycosylases OGG1 and MUTYH in the BER system are essential proteins in the repair of ROS-induced DNA oxidative damage. The BER pathway is the main mechanism for the removal of DNA base lesions to prevent consequential mutagenesis. ROS levels in cells may be elevated under oxidative stress due to physiological changes and environmental exposures [19]. Our results showed enhanced 8-oxoG staining in BLM-induced pulmonary fibrosis models. Accumulating evidence demonstrated a genetic susceptibility to mutation-related diseases due to BER gene defects. Farrington et al. reported that individuals with MUTYH defects had a significantly increased risk of colorectal cancer [20], and OGG1 deficiency increased the susceptibility to lung cancer and metabolic dysfunction [21, 22]. Cheresh et al. showed that *Ogg1* knockout mice exhibited augmented asbestos-induced pulmonary fibrosis [23]. However, we previously described that the impact of MUTYH deficiency was based on the degree of oxidative stress and was tissue-dependent. Wild-type mice exhibited more serious lung lesions than *Mutyh*^−/−^ mice with D-gal-induced oxidative stress [14], and the current study provides supporting data. We observed an attenuated pulmonary fibrosis phenotype in BLM-treated *Mutyh*^−/−^ mice compared to *Mutyh*^+/+^ mice. To understand this result, we used immunohistochemistry to detect 8-oxoG in the pulmonary tissues of model mice and found no significant difference in the 8-oxoG staining index between *Mutyh*^+/+^ and *Mutyh*^−/−^ mice treated with BLM, which suggests that MUTYH deficiency induced no discernible additional oxidative DNA damage in BLM-induced pulmonary. Given that BER initiated by MUTYH generates AP sites, which can lead to the buildup of SSBs with severe oxidative stress, we further revealed more accumulation of SSBs in pulmonary tissues of *Mutyh*^*+*/*+*^ than *Mutyh*^−/−^ mice treated with BLM in the late stage of fibrogenesis. Oka, et al. also reported that MUTYH deficiency substantially prevents the formation of SSBs in mtDNA of striatum neurons exposed to 3-nitropropionic acid [9]. The depletion of mtDNA may be attributable to the accumulation of SSBs, which can also trigger caspase-independent/calpain-dependent cell death [8]. The current result in our study showed an increased amplification efficiency of long-range PCR of the mtDNA fragment in BLM-treated *Mutyh*^−/−^ mice of the D14 and D28 groups when compared to the counterpart of *Mutyh*^+/+^. The lack of a significant difference in mtDNA content in pulmonary cells between BLM-*Mutyh*^+/+^ and BLM-*Mutyh*^−/−^ D14 and D28 mice suggests that MUTYH deficiency is favorable for the maintenance of mtDNA integrity under severe oxidative stress. We also performed morphological observations of mitochondria using electron microscopy and examined the expression of proteins for mitochondrial dynamics. We detected a higher mitochondrial number in AECIIs of NS-*Mutyh*^−/−^ and BLM-*Mutyh*^−/−^ mice in the D7 group and an increased expression of the mitochondrial fission protein DRP1 and the mitophagy protein PINK1 in pulmonary tissues of BLM-*Mutyh*^−/−^ mice in the D14 and/or D28 groups. These proteins control mitochondrial quality and protect AECIIs from apoptosis. A MUTYH deficiency-associated attenuation of epithelial apoptosis was observed in model animals and in *in vitro* experiments in the current study.

Notably, increased levels of the profibrotic cytokine TGF-*β*1 were observed in pulmonary tissues of *Mutyh*^−/−^ mice treated with intratracheal saline compared to wild-type mice at D7. Our recent study also showed reduced SOD activity in lung tissue of *Mutyh*^−/−^ mice that received a hypodermic injection of normal saline, as a control group, in an oxidative stress experiment [14]. This result suggests that MUTYH deficiency affects ROS levels in lung cells following a slight stimulation. Cumulatively, it is plausible that MUTYH deficiency aggravates oxidative damage under NS stimulation, especially in the acute inflammation period. We previously described the novel variation *AluYb8MUTYH*, an *AluYb8* element insertion in the 15th intron of the human *MUTYH* gene. This variation greatly reduced MUTYH type 1 protein (MUTYH1) in mitochondria. We recently reported that *AluYb8MUTYH* impaired mtDNA stability and affected the age of onset of IPF, but it did not increase susceptibility to pulmonary fibrosis [24]. Combining with the results of this study, we consider that MUTYH deficiency has an adverse impact on health because it can give rise to elevated spontaneous mutations in physiological aging under mild oxidative stress. On the other hand, MUTYH deficiency-associated BER suppression reduced the buildup of DNA SSBs, which induced cell death and contributed to the maintenance of cell survival and the attenuation of tissue lesions under severe oxidative stress.

## 5. Conclusions

In summary, we demonstrated that MUTYH deficiency was associated with attenuated pulmonary fibrosis in BLM-induced mice. MUTYH deficiency prevented the buildup of DNA single-strand breaks and maintained mtDNA integrity in pulmonary tissue cells, which contributed to AECII survival. The current study suggests MUTYH inhibition as a new therapeutic approach to protect pulmonary tissue under severe oxidative stress.

## Data Availability

Data are available upon request.

## Supplementary Materials

Supplementary Materials Figure S1: serum TGF-*β*1 levels of bleomycin- (BLM-) induced mice. Figure S2: the relative content of mtDNA in pulmonary tissue cells of model animals. Figure S3: the relative expression levels of MUTYH in A549 cells with RNA interference. Figure S4: inhibition of MUTYH expression was associated with reduction of A549 cell apoptosis under oxidative stress. Table S1: siRNA sequences. Table S2: primer sequences.
