# Supplementary material for: MUTYH Deficiency Is Associated with Attenuated Pulmonary Fibrosis in a Bleomycin-Induced Model
Source: Oxid Med Cell Longev. 2020 Oct 16;2020:4828256. doi: 10.1155/2020/4828256 (PMC7603627; doi:10.1155/2020/4828256)
Supplement: Supplementary Materials — Figure S1: serum TGF-β1 levels of bleomycin- (BLM-) induced mice. Figure S2: the relative content of mtDNA in pulmonary tissue cells of model animals. Figure S3: the relative expression levels of MUTYH in A549 cells with RNA interference. Figure S4: inhibition of MUTYH expression was associated with reduction of A549 cell apoptosis under oxidative stress. Table S1: siRNA sequences. Table S2: primer sequences. [file 4828256.f1.docx]

**Supplementary data**

**MUTYH deficiency is associated with an attenuated pulmonary fibrosis in bleomycin-induced model**

Qingmin Sun ^a,b^, Jingwen Chen^a,d^, Lizhi Xu^a,d^, Jiaqi Kang^a,d^, Xin Wu^a,d^, Yan Ren^a,d^, Yusaku Nakabeppu ^c^*, Yaping Wang^a,d,^*

^a^ Department of Medical Genetics, Nanjing University School of Medicine, Nanjing 210093, China

^b^ Department of Pharmacy, Jiangsu Province Hospital of Chinese Medicine, Affiliated Hospital of Nanjing University of Chinese Medicine, Nanjing, Jiangsu 210029, China

^c^ Division of Neurofunctional Genomics, Department of Immunobiology and Neuroscience, Medical Institute of Bioregulation, Kyushu University, Fukuoka 812-8582, Japan

^d^ Jiangsu Key Laboratory of Molecular Medicine, Nanjing University, Nanjing 210093, China

**Corresponding author*: Department of Medical Genetics, Medical School, Nanjing University, Nanjing 210093, China. E-mail addresses: wangyap@nju.edu.cn (Y. Wang). Division of Neurofunctional Genomics, Department of Immunobiology and Neuroscience, Medical Institute of Bioregulation, Kyushu University, Fukuoka 812-8582, Japan. E-mail address: [yusaku@bioreg.kyushu-u.ac.jp](mailto:yusaku@bioreg.kyushu-u.ac.jp) (Y. Nakabeppu).

**
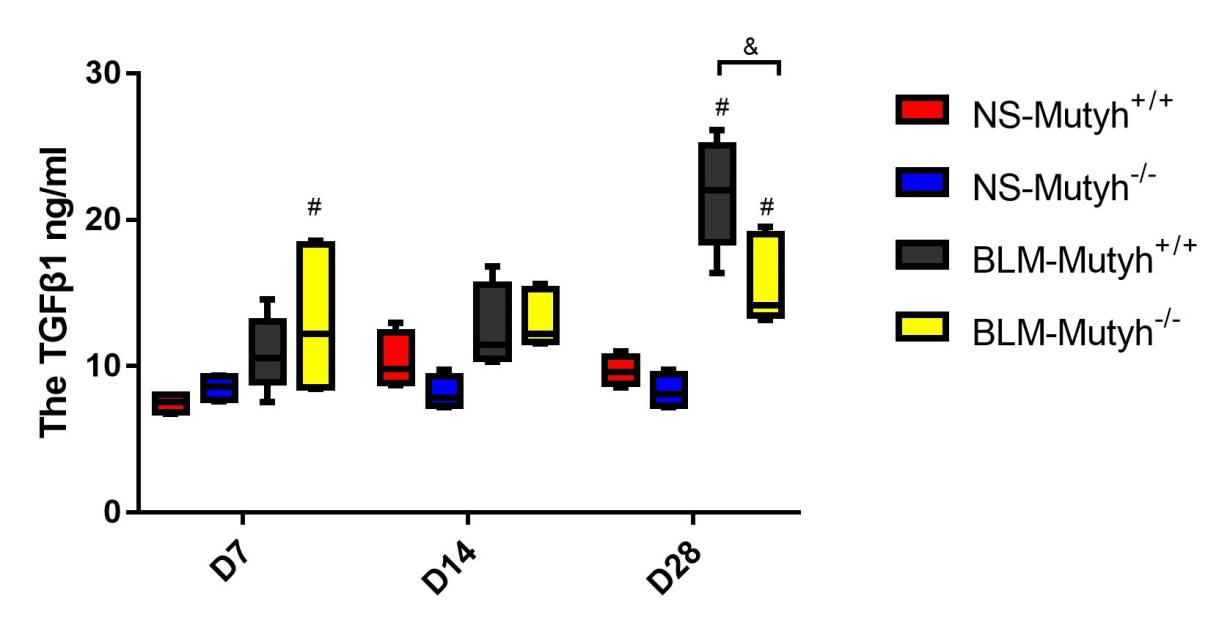
**

**Supplementary Fig. S1. Serum TGF-β1 levels of bleomycin (BLM)-induced mice.** Mice serum TGF-β1 levels were determined by Elisa kit, showing the contents of TGF-β1 in Mutyh^+/+^ mice were higher than that of Mutyh^-/-^ animals at day 28 (P < 0.001) (n ≥ 5 mice, mean ± SEM). Statistical significance was analyzed using two-way ANOVA and followed by LSD post-hoc test. *represents P < 0.05, ^#^represents P < 0.01 compared with NS-Mutyh^+/+^ group. ^&^represents P < 0.01 compared to BLM-*Mutyh*^+/+^ group


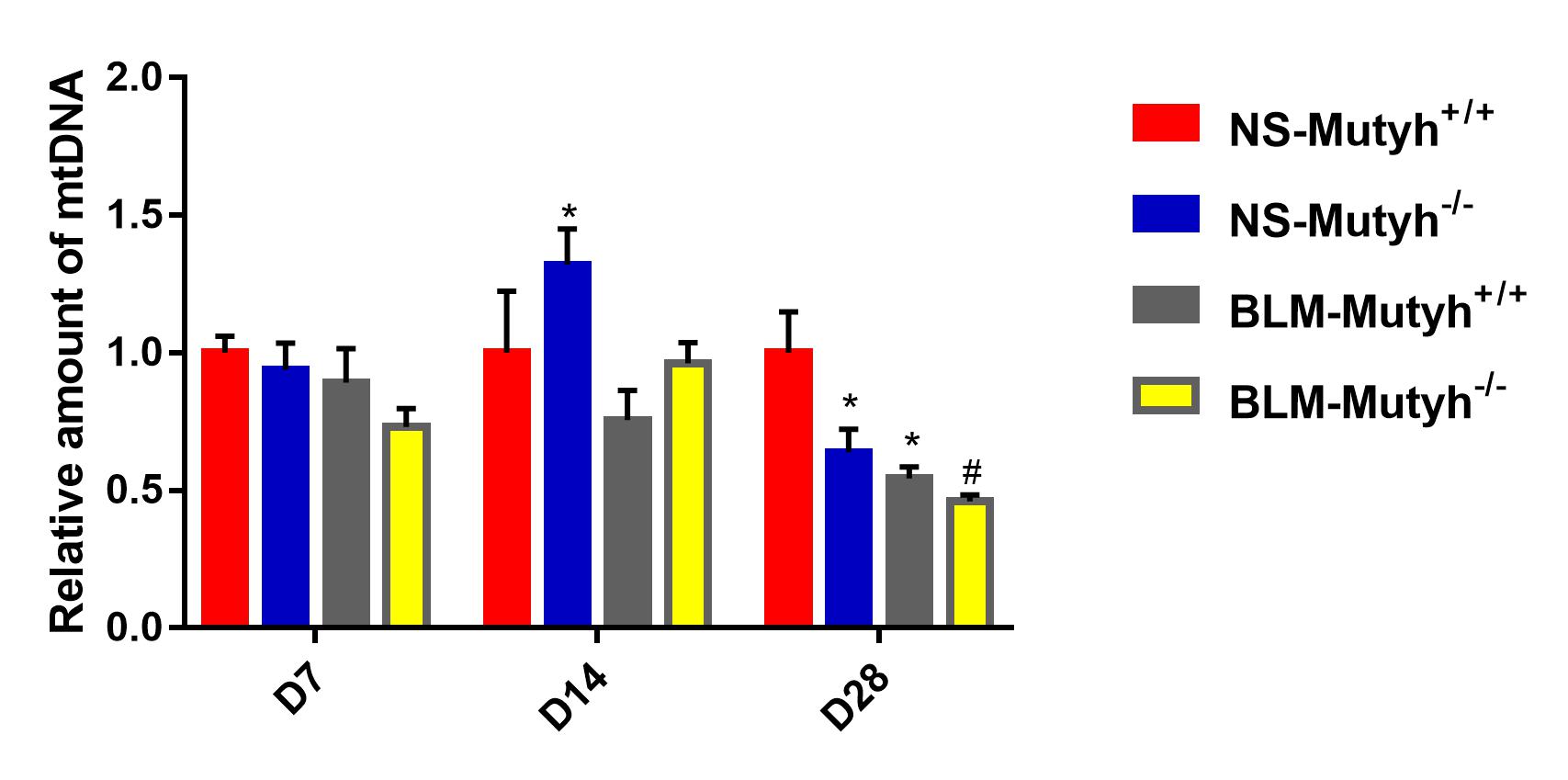


**Supplementary Fig. S2. The relative content of** **mtDNA in pulmonary tissue cells of model animals.** Quantitative results of mtDNA copy numbers in pulmonary tissue cells of the model animals. The values represent the ratio of the 117 bp fragment in mtDNA to β-actin fragment of nDNA (n ≥ 5). Statistical significance was analyzed using two-way ANOVA and followed by LSD post-hoc test. *represents P < 0.05, #represents P < 0.01 compared with NS-Mutyh^+/+^ group.


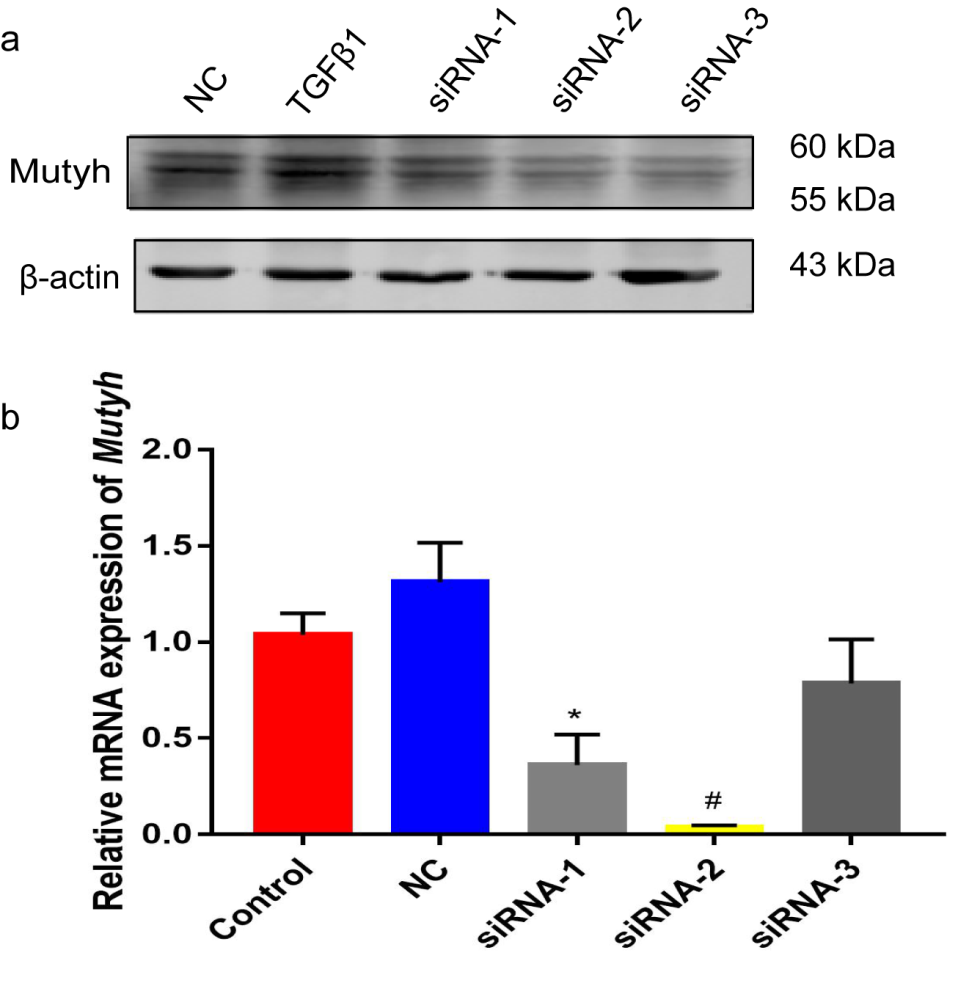


**Supplementary Fig. S3. The relative expression levels of MUTYH in A549 cells with RNA interference.** The human alveolar type II pulmonary epithelium A549 cells were transfected with siRNA-MUTYH. (a) Western blotting showed that the expression of MUTYH protein decreased significantly with siRNA-2 transfection. (b) Q-PCR indicated that siRNA-2 significantly inhibited MUTYH mRNA expression compared with scrambled siRNA control (P = 0.002)(n = 3, mean ± SEM). Statistical significance was analyzed using one-way ANOVA and followed by LSD post-hoc test. *represents P < 0.05, ^#^represents P < 0.01 compared with NC (negative control).

**
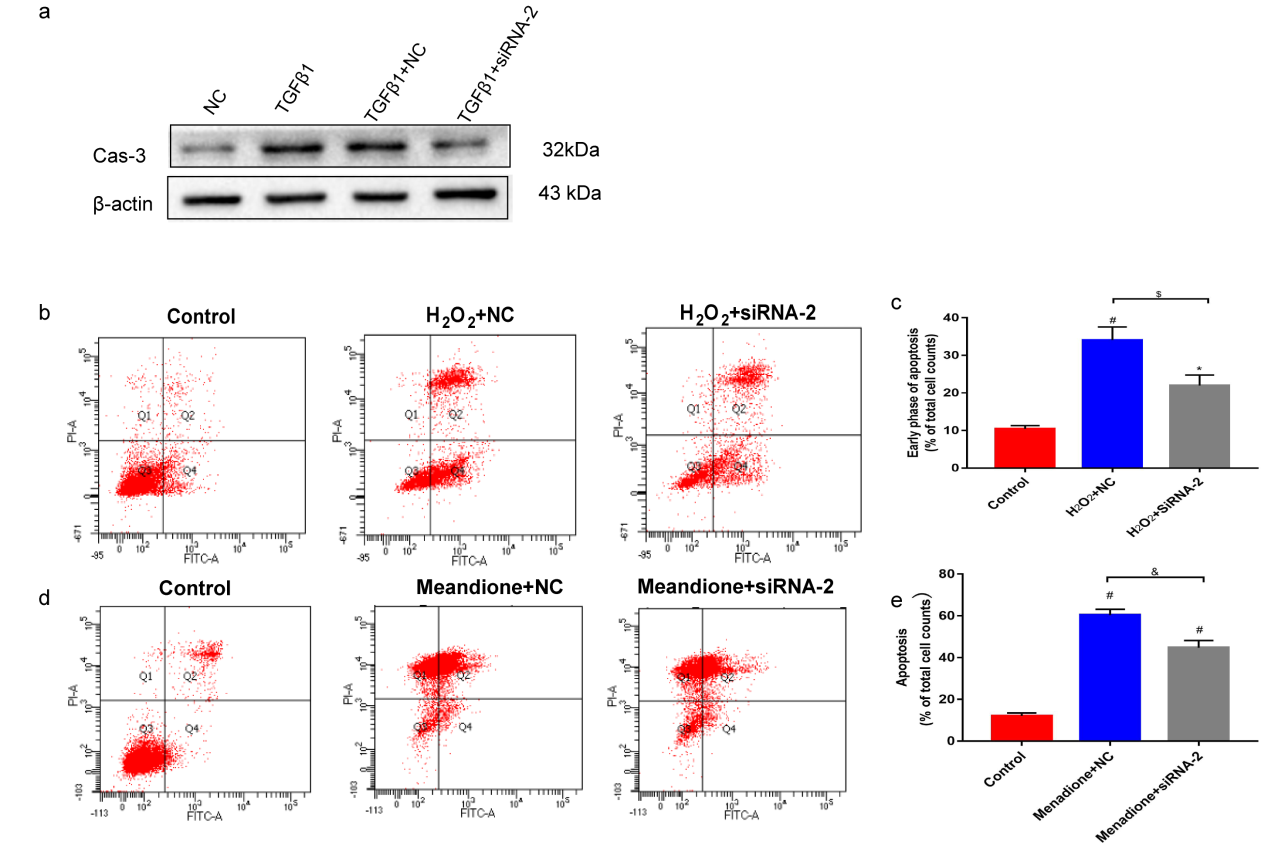
**

**Supplementary Fig. S4. Inhibition of MUTYH expression was associated with reduction of A549 cells apoptosis under oxidative stress** (a) The expression of Caspase-3 proteinin TGF-β1 stimulated A549 cells with MUTYH RNA interference. . WB showed that expression of TGF-β1-induced Caspase-3 was inhibited with MUTYH siRNA transfection. (b-e) A549 cells were incubated with 500uM H_2_O_2_ or 500uM menadione for 4 h, and then cell apoptosis was measured using Annexin V-FITC/PI Kit. (b-c) Compared with control, MUTYH knockdown with siRNA could significantly repress early phase of apoptosis when A549 cells were incubated H_2_O_2_ (P = 0.010). (d-e) When A549 cells treated with menadione, the results also showed that siRNA was associated with a decreased apoptotic cells (P = 0.002) (n = 4, mean ± SEM). Statistical significance was analyzed using one-way ANOVA and followed by LSD post-hoc test. *represents P < 0.05, ^#^represents P < 0.01 compared with control. $P < 0.05, & P < 0.01.

**Supplementary Table S1.** siRNA sequences

| Name | Sequence (5’ to 3’) |
| --- | --- |
| MUTYH-1-sense  MUTYH-1-antisense  MUTYH-2-sense | GGAGGCAGAAGCAUGCUAATT  UUAGCAUGCUUCUGCCUCCTT  GCAUAUGCUGUGUGGGUCUTT |
| MUTYH-2-antisense | AGACCCACACAGCAUAUGCTT |
| MUTYH-3-sense  MUTYH-3- antisense  Negative Control-sense | GCCAGGAGAUUUCAACCAATT  UUGGUUGAAAUCUCCUGGCTT  UUC UCC GAA CGU GUC ACG UTT |
| Negative Control -antisense | ACG UGA CAC GUU CGG AGA ATT |

**Supplementary Table S2.** Primer sequences

| Name | Sequence (5’ to 3’) |
| --- | --- |
| mt-117bp-Forward | CCCAGCTACTACCATCATTCAAGT |
| mt-117bp-Reverse | GATGGTTTGGGAGATTGGTTGATG |
| 10kb-Forward | GCCAGCCTGACCCATAGCCATAATAT |
| 10kb-Reverse | GAGAGATTTTATGGGTGTAATGCGG |
| 8.7kb-Forward | TTGAGACTGTGATTGGCAATGCCT |
| 8.7kb-Reverse | CCTTTAATGCCCATCCCGGAC |
| MUTYH-Forward | GTGTGTATCAGGGCCAACAG |
| MUTYH-Reverse | ACAGGATTCTCAGGGAATGG |
| β-actin-Forward | GTACCACCATGTACCCAGGC |
| β-actin-Reverse | GCAGCTCAGTAACAGTCCGC |
